# An Open Letter to Private Nurses

**Published:** 1920-12-25

**Authors:** Geraldine Bremner

**Affiliations:** 63 Wimpole Street


*To the Editor of* The Hospital.

### An Open Letter to Private Nurses.

*To the Editor of* The Hospital.

Sir,—May I through your columns address this letter to my fellow-sisters : As the General Nursing Council are asking Dr. Addison to introduce a Bill to regulate the hours of nurses, I should be very glad, as a representative of private nurses on the Council of the College of Nursing and on the Committee of the Nurses' Co-operation, to have the opinion of my colleagues on the subject of their inclusion in the proposed Bill. I am therefore asking all private nurses who may see this letter to write to me at once to say whether they wish their hours regulated by the State (viz., in Dr. Addison's Bill).

In my opinion, should a Bill as stated be introduced by the Minister, it should include every branch of nursing. Private nurses should not be left out, as has been suggested. In fact, I think such a procedure would do them incalculable harm.

A Bill brought in by the State to regulate the hours of

**The Editor's Letter Box—*(continued)*.**

all nurses will do more to impress the public mind that nurses are not machines and cannot work unlimited hours than anything else will.

We may rest assured that such a Bill will be drafted or regulated in such a manner that it will inflict no hardship on patients. Probably private nurses would be free to work longer hours during the critical stage of illness, and hours of work be shorter during convalescence. In my capacity as their representative I wish when opportunity arises to voice the opinion of the majority, and it is for this reason I ask them to communicate with me, and as a token of good faith to put the initials of any nursing society they may belong to after their signatures.—Yours faithfully, GERALDINE BREMNER.

63 Wimpole Street, December 19, 1920.

